# High-speed III-V nanowire photodetector monolithically integrated on Si

**DOI:** 10.1038/s41467-020-18374-z

**Published:** 2020-09-11

**Authors:** Svenja Mauthe, Yannick Baumgartner, Marilyne Sousa, Qian Ding, Marta D. Rossell, Andreas Schenk, Lukas Czornomaz, Kirsten E. Moselund

**Affiliations:** 1 IBM Research Europe, Säumerstr. 4, 8803 Rüschlikon, Switzerland; 2 Department of Information Technology and Electrical Engineering, Integrated Systems Laboratory, ETH Zurich, Gloriastr. 35, 8092 Zurich, Switzerland; 3 Electron Microscopy Center, Empa, Swiss Federal Laboratories for Materials Science and Technology, Überland Str. 129, 8600 Dübendorf, Switzerland

**Keywords:** Materials for optics, Nanoscale devices, Applied optics, Electronics, photonics and device physics

## Abstract

Direct epitaxial growth of III-Vs on silicon for optical emitters and detectors is an elusive goal. Nanowires enable the local integration of high-quality III-V material, but advanced devices are hampered by their high-aspect ratio vertical geometry. Here, we demonstrate the in-plane monolithic integration of an InGaAs nanostructure *p-i-n* photodetector on Si. Using free space coupling, photodetectors demonstrate a spectral response from 1200-1700 nm. The 60 nm thin devices, with footprints as low as ~0.06 μm^2^, provide an ultra-low capacitance which is key for high-speed operation. We demonstrate high-speed optical data reception with a nanostructure photodetector at 32 Gb s^−1^, enabled by a 3 dB bandwidth exceeding ~25 GHz. When operated as light emitting diode, the *p-i-n* devices emit around 1600 nm, paving the way for future fully integrated optical links.

## Abstract (web summary)

Direct epitaxial growth of III-V on Si for optical emitters and detectors remains a challenge. Here, the authors demonstrate in-plane monolithic integration of an InGaAs nanostructure *p-i-n* photodetector on Si capable of high-speed optical data reception at 32 Gbps enabled by a 3 dB bandwidth exceeding 25 GHz.

## Introduction

Data traffic and integration density in electronic integrated circuits has increased drastically in the past decade, resulting in an increase in power consumption of computing resources worldwide^1–3^. A large fraction of this power is used to transmit data along resistive electrical interconnects. It has long been the goal to integrate optical interconnects on the electronic chips itself, and recent progress in scaling and performance of integrated photonic components has made this vision more tangible. To present a viable alternative to copper interconnects, optical links need to provide significantly better energy efficiency, while supporting higher bandwidth densities and lower cost per bit. This requires physically scaled components with ultra-low capacitance that are densely integrated with their control electronics by leveraging monolithic integration technologies^4^. While some optical link schemes are based on off-chip lasers^5,6^, others foresee the integration of multiple lasers or light emitting diodes (LEDs) on chip^7^. In both cases, densely integrated high-speed photodetectors are key components for on-chip optical transceivers. State-of-the-art integrated photodetectors are commonly based on germanium, since this material is readily available in CMOS foundries to enhance hole mobility in compressively strained *p*-channel transistors. Scaled Ge photodetectors have reached a high level of maturity, with detection bandwidths exceeding 70 GHz and operation at 100 GBd^8,9^, however, they often tend to suffer from high dark currents and low absorption due to the indirect nature of Ge. III–V materials on the other hand, exhibit higher absorption coefficients and due to their direct tunable bandgap, they also enable active optical emitters such as lasers^10–13^ or LEDs^14–16^. The integration on Si, however, is significantly more challenging because of the substantial lattice, polarity, and thermal mismatch. A seamless integration scheme between scaled photonic and electronic components will require a local monolithic integration approach. Nanowire (NW) techniques are of interest due to their small epitaxial interface with Si which allows for the growth of high-quality single crystalline material despite a high degree of lattice mismatch. Furthermore, the small size of NW detectors results in a capacitance in the sub-fF regime, which is required for both low-power and high-speed operation. Different fabrication techniques have been pursued from growing III–V NWs on III–V substrates^17–19^ to catalyzed growth on Si using Au particles^20,21^ or selective area III–V growth on Si^15,22–24^. Existing NW technologies however, either result in vertically oriented devices^15,18,23^ or when integrated laterally, i.e., lying on the substrate, are based on pick-and-place techniques^17,19,21^. So far, neither of these approaches are suitable for large-scale integration or waveguide coupling, nor are they compatible with conventional CMOS process flows. Moreover, to the best of our knowledge, there has only been a single demonstration of NW detectors monolithically integrated on Si operating in the GHz regime. In ref. ^15^, Chen et al. demonstrated an InGaAs core–shell *p–i–n* structure with a 3 dB bandwidth of 3.1 GHz, a factor of 10 lower than what is required for optical interconnects.

In this work, we demonstrate the first ultrathin and monolithically integrated nanostructure *p–i–n* photodetector on Si with a responsivity reaching ~0.68 A W^−1^ in the O-band (1260–1360 nm). We demonstrate for the first time high-speed optical data reception at 32 Gb s^−1^ and a 3 dB bandwidth exceeding ~25 GHz using a nanostructure photodetector, as well as photodetection in the C-band (1530–1565 nm). Moreover, when operated as an LED, the fabricated *p–i–n* devices show light emission in the C-band, thus paving the way for a fully integrated optical link.

## Results

### Integration of III–V photonic devices on Si using TASE

**Fig. 1:**
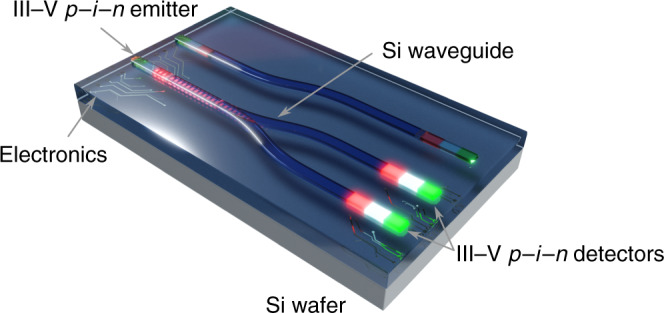
Vision of a future all-optical link fabricated via TASE. Both emitter and detector can be integrated in-plane and butt-coupled to a Si waveguide. The present work employs free-space coupling but paves the way for future waveguide-coupled devices.

**Fig. 2:**
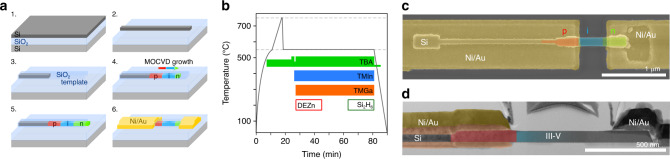
Template-assisted selective epitaxy fabrication process. **a** Process steps during TASE. 1. SOI wafer. 2. Patterned top Si layer. 3. Partially hollow SiO_2_ template with Si seed. 4. MOCVD growth of *p–i–n* InGaAs. The arrow indicates the growth direction. 5. *p–i–n* nanostructure after growth. 6. Ni/Au contacts on nanostructure. **b** Schematic of the *p–i–n* InGaAs MOCVD growth sequence. The colored regions correspond to the turning on of the flow of precursors. First, an annealing step is performed to desorb water residues. Next, a short InAs seed is deposited for nucleation followed by the *p–i–n* InGaAs growth. Details can be found in the Supplementary Note 1. **c** False-colored SEM top view of *p–i–n* nanostructure. **d** Bright field STEM image of a cross-section of the nanostructure in (**c**). Colors indicate materials in the left part.

Figure 1 shows our vision of a future III–V based electro-optical platform on Si with optical devices, such as photodetector and light source, directly coupled to a Si waveguide. To make this platform a reality, III–V materials have to be integrated directly on Si, in-plane with silicon features and with lateral in-situ doping profiles to enable device operation. In this work, we propose a novel device integration scheme that enables in-plane junctions and direct growth of III–V material on Si waveguides. Devices are grown on Si and characterized using free-space optical coupling, as current dimensions of the silicon waveguide onto which the *p–i–n* structure is grown are too small to support a propagating optical mode. A key feature of our devices is an in-plane doping profile resulting in a horizontal *p–i–n* nanostructure compared to commonly used vertical or radial *p–i–n* stacks. Using template-assisted selective epitaxy (TASE^25^) combined with in-situ doping, we integrated InGaAs homojunctions epitaxially on Si. The in-plane approach may allow for easy waveguide coupling in the future by directly growing the III–V active material as an extension to SOI waveguides. This technique has previously been used to demonstrate in-plane electronic devices^26^ and optically pumped microdisks^12^. The concept of TASE relies on an oxide template to guide the epitaxial growth as illustrated in Fig. 2a. The top Si layer of an SOI wafer is patterned, covered with SiO_2_ forming an oxide template, and the Si partially etched back through an opening in the SiO_2_ to form a hollow template. III–V material is grown from the Si seed into the hollow SiO_2_ template. Structures can be densely integrated since the location of the III–V is defined lithographically in the same step as the silicon passives. Moreover, the technique offers co-integration of multiple III–Vs^27^, heterojunctions^28^, and the possibility of integrating quantum wells. The fabrication is performed on standard SOI substrates with 2 μm buried oxide (BOX). More details on the fabrication can be found in the “Methods” section. In this work, nanostructure shaped SiO_2_ templates are formed following the fabrication steps depicted in Fig. 2a. Starting at the Si seed, InGaAs is grown into the hollow oxide template using metal-organic chemical vapor deposition (MOCVD) at 550 °C. In-situ *p*- and *n*-doped regions are achieved by adding doping precursors during the growth and their extension controlled by the growth duration. Starting at the Si seed, ~330 nm *p*-doped InGaAs are grown, followed by ~330 nm intrinsic InGaAs, and finally ~330 nm *n*-doped InGaAs (the growth sequence is shown in Fig. 2b and more detail is provided in the Supplementary Notes 1 and 2). The final *p–i–n* InGaAs nanostructures exhibit a length of ~1 μm, a height of 60 nm, and widths between 60 nm and 500 nm as defined in the design. Electrical contacts are formed using a Ni/Au lift-off process. Figure 2c and d show an SEM image of an as grown and contacted device and a cross-section along the center of the nanostructure, respectively. An activation energy of ~200 meV is measured on the devices (see Supplementary Note 6) suggesting a small energy barrier at the *p*-type contact. With resulting ultra-small footprints as low as ~0.06 μm^2^, the fabricated *p–i–n* nanostructures are excellent candidates for densely integrated Si PICs. The scaled dimensions of the device suggest a junction capacitance below 0.1 fF.

### Detailed material characterization

**Fig. 3:**
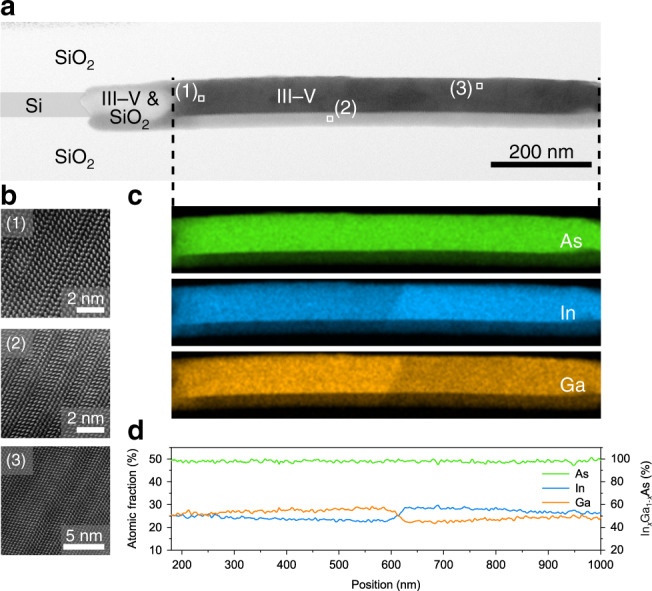
Epitaxial single crystalline InGaAs nanostructure. **a** Bright field STEM overview image of a 200 nm wide *p–i–n* nanostructure without contacts. The III–V material is grown from the Si in a funnel shape and hence, results in a thinner III–V region overlaid with SiO_2_ at the Si interface which is visible as a brighter region. The Si seed is false colored to enhance visibility. **b** High resolution annular dark field STEM images taken at marked positions in (**a**). **c** EDS analysis of the different elements in the In_*x*_Ga_1−*x*_As nanostructure. **d** Extracted atomic fraction along the nanostructure. Compositional line profiles obtained by averaging the integrated intensities over the nanostructure diameter.

To investigate the material quality, scanning electron microscopy (SEM), scanning transmission electron microscopy (STEM), and energy dispersive X-ray spectroscopy (EDS) analysis are performed on several focused ion beam prepared lamellas containing a cross-section of a nanostructure (see Fig. 3a). The STEM analysis reveals a single crystalline structure epitaxially aligned with the Si seed crystalline orientation with misfit dislocations locally confined to the Si/III–V interface. High resolution images depicted in Fig. 3b demonstrate the absence of threading dislocations in this device but reveal stacking faults and short wurtzite (WZ) segments in the zincblende (ZB) InGaAs structure. Figure 3c shows the EDS elemental maps of As, In, and Ga along the nanostructure, revealing an average In_*x*_Ga_1−*x*_As concentration of *x*~50% (see Fig. 3d). Moreover, small variations in the In content between *x*~47% and *x*~53% are observed corresponding to unstrained theoretical ZB bulk bandgaps of *E*_G_(In_0.47_Ga_0.53_As)~0.78 eV and *E*_G_(In_0.53_Ga_0.47_As)~0.74 eV, respectively. We believe that the composition variation can be correlated with the switching of doping precursors during MOCVD growth. This is consistent with previous work where similar effects were observed^29,30^.

### In-plane nanostructure photodetectors

**Fig. 4:**
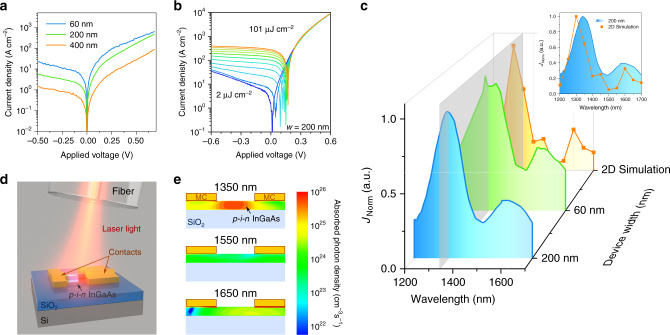
Static electro-optical characterization of the *p–i-n* InGaAs photodetectors. **a** *I*–*V* curves for devices with different widths. **b** *I*–*V* curves for increasing incident laser powers (1346 nm) of the 200 nm wide device. **c** Normalized spectral response for devices with different widths at −0.5 V bias as well as for a simulated device (2D). The current density is normalized for better visibility, however the current density for individual devices is in the same order of magnitude of 100 A cm^−2^. The inset depicts a comparison of the experimentally obtained spectral response for a 200 nm wide device and the simulated 2D device using electro-optical simulations. **d** Schematic of the free-space coupling for electro-optical devices. **e** Simulated absorbed photon density for different incident wavelengths (1350, 1550, and 1650 nm) on *p–i–n* InGaAs devices with metal contacts (MC). The devices are 60 nm high and 1 µm long. The *z*-direction is enlarged by a factor of 2 for better visibility.

Photodetectors are characterized in different optical set ups to evaluate their spectral response and high-speed operation. Extensive numerical simulation using “Synopsys Sentaurus”^31^ is used to analyze their behavior. First, the dark current performance of the fabricated photodetectors is characterized without optical illumination (dark). A low dark current is desired, enabling a high signal-to-noise ratio. The *p–i–n* nanostructures show diode-like *I*–*V* characteristics as depicted in Fig. 4a. Devices of 60 nm width exhibit dark currents in reverse bias as low as 1.7 nA at −2 V, for devices with 200 nm and 500 nm width dark currents of 15 nA and 36 nA at −2 V were measured, respectively (more details in Supplementary Note 5). These values compare well with dark currents measured in bonded, high material quality III–V photodetectors^32^ as well as in conventional InGaAs photodetectors^33^ and are lower than values achieved in grown InGaAs metal–semiconductor–metal NW photodetectors^20^. To investigate the spectral response of the photodetectors, devices are illuminated with a ps-pulsed supercontinuum laser (78 MHz repetition rate) and their *I*–*V* characteristics measured. Figure 4b depicts *I*–*V* curves of a single 200 nm wide detector with increasing incident ps-pulsed optical power at 1346 nm. Next, the incident laser wavelength is varied between 1000 and 1750 nm. Figure 4c compares the current density for different device widths normalized to their respective maximum. The spectral responses of the devices are similar with light absorption between 1200 and 1700 nm and a maximum around 1350 nm. A flat spectral response as for bulk InGaAs is not expected due to the combination of the small device thickness and the free-space coupling, which favors the absorption of shorter wavelengths closer to the surface. To further understand the spectral response of the devices we perform 2D electro-optical simulations using “Synopsys Sentaurus” simulation software. A device structure as depicted in Fig. 4d is simulated with monochromatic light impinging under 10° corresponding to the free-space light coupling in the experimental setup. The simulations reveal multiple reflections of the incident light between the Si substrate, the SiO_2_ BOX layer, and within the device between its contacts (see Supplementary Note 9). This results in an incident wavelength dependent optical generation in the structure as depicted in Fig. 4e and hence, a spectral response with two distinct absorption peaks around 1350 and 1650 nm (Fig. 4c). This simulated spectral response confirms the experimentally measured data.

An important measure to characterize a photodetector is its efficiency to convert incident photons to electron–hole pairs, namely the responsivity. Whereas, the responsivity is easily measured in a fully integrated platform, in the case of NWs or nanostructures it can be calculated based on the measured photocurrent and an estimation of the incident illumination power^21,24^. To determine the responsivity at the maximum absorption, we excite the *p–i–n* structures with a continuous wave (cw) laser at 1346 nm. The incident light intensity is calculated taking the position of the optical fiber and the illuminated device area into account (see details in Supplementary Note 10 along with the bias-dependent responsivity). The measured device shows a responsivity up to 0.68 A W^−1^ at −2 V and a quantum efficiency up to 62%. If we assume that carriers generated in the doped regions also contribute to the photocurrent, the calculated responsivity drops to ~0.2 A W^−1^. These values are very promising and compare well with values achieved in similar InGaAs structures^15^. A detailed analysis of the responsivity can be found in the Supplementary Note 10.

### Electrically and optically pumped light emission

**Fig. 5:**
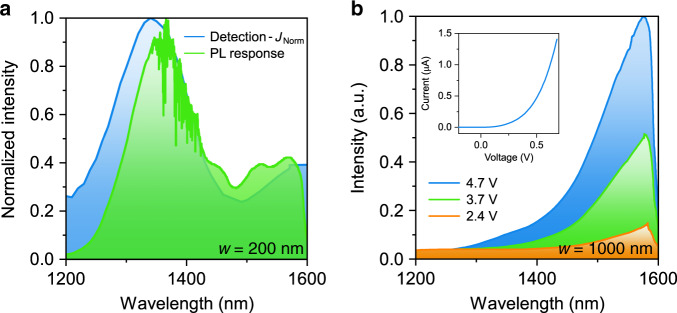
Electrically and optically pumped light emission. **a** Measured PL and spectral absorption response spectra (*w* = 200 nm). **b** EL from a ~1 μm wide device for different applied voltages. Close to 1600 nm, the cutoff of the measurement detector connected to the spectrometer is visible. The inset shows the *I*–*V* curve in forward bias.

To further investigate the optical spectrum of the *p–i–n* structures, photoluminescence (PL) spectroscopy is performed and the obtained spectrum depicted in Fig. 5a. The spectrum reveals an emission peak around ~1600 nm, corresponding to expected values from bulk ZB In_*x*_Ga_1−*x*_As for the determined compositions in Fig. 3d. Moreover, the obtained PL spectrum agrees well with the spectral response of the nanostructure, which is influenced by reflections at the various interfaces. Furthermore, it may be influenced by the relatively high pump powers that are required to get sufficient light output from the nanostructures^34,35^.

Electroluminescence (EL) with a single emission peak in the desired C telecommunication band is observed when devices are operated as an LED in forward bias, as shown in Fig. 5b. With increasing electrical pumping, the intensity of the EL increases. The EL spectrum matches well the second emission peak measured in PL. These initial measurements demonstrate the dual functionality of the *p–i–n* nanostructures for both light detection and emission.

### High-speed operation

**Fig. 6:**
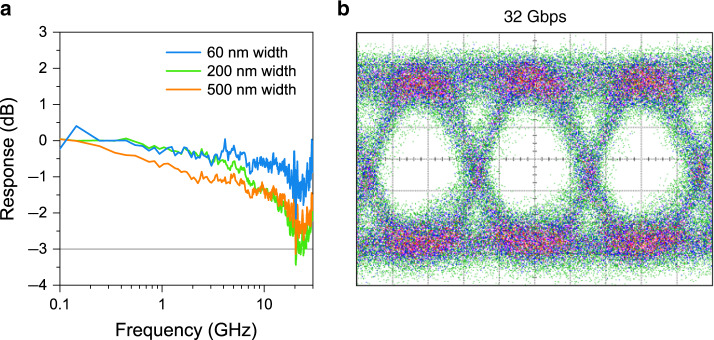
High-speed data reception. **a** *S*_21_ measurements for devices with different widths. The line at −3 dB marks the 3 dB limit. **b** 32 Gb s^−1^ eye diagram measured on a 500 nm wide device at −2 V applied bias (1.5 mV div^−1^, 10 ps div^−1^). Averaging was set to 128.

High-speed operation is key for future applications and one of the main motivations for moving from electronics to optical signal propagation. We measure the frequency response of the photodetectors and determine their 3 dB bandwidth using a modulated optical signal at 1346 nm illuminated from the top. Figure 6a depicts the measured frequency response (scattering parameter *S*_21_) of a 200 nm wide photodetector biased at −1.5 V. The plot reveals a 3 dB bandwidth beyond our measurement capabilities (~25–30 GHz). The demonstrated 25 GHz bandwidth is, to the best of our knowledge, the highest reported value for nanostructure photodetectors. To further evaluate the performance at high baud rates, we acquire electrical eye-diagrams. A non-return-to-zero on–off Keying pseudo random binary sequence of 2^7^−1 bits is sent onto the photodetectors and their electrical response measured on a sampling scope. Figure 6b depicts an eye diagram obtained at 32 Gb s^−1^. A clear eye opening at high data rates is visible, confirming the high-speed nature of our photodetectors.

## Discussion

In conclusion, the lateral integration enabled by TASE permits the in-plane integration of homo- and heterojunctions within a nanostructure geometry. The seamless integration with silicon along with the in-plane profiles, which are more easily compatible with conventional processing, are essential for future VLSI electronic-photonic circuits on-chip. The monolithically integrated InGaAs *p–i–n* photodetectors can cover several telecommunication bands, with a maximum responsivity up to 0.68 A W^−1^ at 1346 nm. Furthermore, detection bandwidths exceeding 25 GHz were demonstrated, enabling high-speed operation at 32 GBd. TCAD simulations are used to analyze device behavior, the simulated spectral response matches the experimental one, with two peaks at around 1350 and 1600 nm. We believe that in a future waveguide-coupled approach with device diameters supporting a single mode, the performance will be comparable or better due to a strong light confinement in the waveguide structure and the potential for implementing a longer absorption region.

When operated as an LED, EL in the C-band is observed. The presented work highlights the great potential for dense integration of III–V electro-optical devices on silicon, which paves the way for the future integration with Si CMOS electronics, and hence will fuel applications such as energy efficient and high-speed optical interconnects for intra-chip or chip-to-chip communication, advanced image sensing, laser radar (LiDAR), or integrated neurophotonics. Since this technique relies on the direct growth extended in-plane from Si features, future devices can be grown directly from a Si waveguide enabling a full optical link with direct coupling.

## Methods

### Sample preparation

In a first step, the top Si layer of an SOI wafer is thinned down to 60 nm defining the height of the final structures. Next, dimensions, shape, and location of the final structures are lithographically defined and patterned using e-beam lithography and an inductively coupled plasma dry etch of the top silicon layer. The patterned Si structures are partially replaced by III–V material in the final step. This one-step lithographic process allows for a precise alignment of III–V and silicon features. In total, 50 nm atomic layer deposition SiO_2_ and 150 nm SiO_2_ using plasma enhanced chemical vapor deposition are deposited covering the patterned Si structures and serving as an oxide template for the final III–V growth. Openings are locally etched into the SiO_2_ layer partially exposing the underlaying patterned Si structures at one end. In a subsequent tetramethylammonium hydroxide wet etch, the patterned Si is partially etched resulting in a hollow oxide template with a remaining Si seed at one extremity. The wet etch process allows for a precise control of the etch-back distance and results in a smooth surface at the Si where the growth will nucleate. Finally, the hollow SiO_2_ cavity is filled with epitaxial III–V material using MOCVD growth at 550 °C: a small ~20 nm long region of indium arsenide (InAs, precursors: TMIn, TBA) is grown which selectively nucleates at the Si interface. Next, InGaAs is grown using TMIn, TBA, and TBGa precursors with a V/III ratio of 0.27. More details can be found in Supplementary Note 1. By adding dopants, the III–V material can be in situ *p*- or *n*-doped. In this work, DEZn and Si_2_H_6_ are added as *p*- and *n*-dopants, respectively. Details on the doping concentration can be found in the Supplementary Note 2. Contacts were formed using a conventional Ni/Au lift-off process.

### Structural characterization

To analyze the structural quality of the devices, cross-sectional lamellas from five different devices were investigated. The lamellas were prepared using a FEI Helios Nanolab 450S focused ion beam. The cut was performed along the growth direction to reveal the Si seed and its nucleation interface with the III–V material. The lamellas were further investigated by STEM using two different instruments. High resolution images were generated using a double spherical aberration-corrected JEOL JEM-ARM200F microscope operated at 200 kV, while the EDS spectrum image was acquired with a FEI Titan Themis operated at 300 kV and equipped with ChemiSTEM technology. The EDS spectrum image was recorded with a beam current of 1.5 nA, 0.8 nm pixel spacing, and a dwell time of 4 μs pixel^−1^. The elemental maps were calculated from the EDS spectrum image using the In Lα_1_, Ga Kα_1_, and Ga Kα_1_ lines. Additional element maps can be found in Supplementary Note 3.

### Photoluminescence spectroscopy

PL spectroscopy was performed under optical pumping with a ps-pulsed supercontinuum laser (NKT Extreme Red, repetition rate of 78 MHz). Three emission lines of the ps-pulsed supercontinuum laser at 710, 720, and 750 nm are used to optically excite the nanostructures. The pump light was focused onto the devices from the top using a 10× objective. With the same objective, the optical response is collected and free-space coupled to a spectrometer (Princeton SP-2500i) with a cryogenically cooled InGaAs CCD camera (Princeton Instruments, PyLoN-IR, cutoff wavelength of ~1600 nm). The setup enables cooling of the samples down to about 10 K, however, all measurements in the main text are performed at room temperature. Low temperature PL measurements can be found in Supplementary Note 8. A typical device-under test (DUT) is depicted in Supplementary Fig. 2c.

### Electroluminescence spectroscopy

EL measurements are performed in the same setup as PL spectroscopy. To eliminate any thermal damage, a pulse generator is used and a rectangular voltage function is applied with a repetition rate of 10 kHz and a duty cycle of 50%. The EL is again collected from the top using a 10× objective and coupled into the spectrometer. Integration times of 10–50 s are used to obtain a high signal-to-noise ratio.

### Spectral response

The spectral response of the devices is measured using the setup described in the previous sections. A ps-pulsed supercontinuum laser excites the devices from the top (10× objective) and the electrical response is measured using a semiconductor device parameter analyzer (Keysight, B1500A). The excitation wavelength is swept from 900 to 1800 nm. The electrical signal is maximized by adjusting the focus at every measured wavelength.

### Simulations

First, 2D Finite-Difference-Time-Domain (FDTD) optical simulations using “Sentaurus EMW Solver” are performed to produce the optical generation rate profile in the entire *p–i–n* structure. A monochromatic light source with constant optical power of 6 dBm is placed at 2 µm above the device under an incident angle of 10° to normal to mimic the free-space excitation. This optical generation profile is then taken as input in the electrical transport simulation using “Sentaurus Device” to calculate the device current at given excitation wavelengths, however we assume an average incident power rather than a pulsed source. By repeating the simulation flow for different excitation wavelengths (from 1200 to 1700 nm, with a step of 50 nm), the spectral-response curve can be obtained. The simulated *p–i–n* device has a height of 60 nm and a total length of 1.3 µm. Metal contacts and SOI substrate are included in the FDTD simulations, with 4 µm thick air attached to the device on top and at the sides. A constant indium composition of 55% along the *p–i–n* structure is assumed for simplicity. The doping concentration is set to the experimentally measured value (see Supplementary Note 2). Shockley–Read–Hall lifetime for all carriers is set to 1 ns as is well-known for III–V materials, and electron and hole mobility in InGaAs is assumed to be 1100 and 250 cm^2^ V^−1^ s^−1^, respectively.

### High-speed measurements

Responsivity and high-speed measurements were performed in a second optical setup under illumination with an optical fiber (NA 0.14) and a cw laser at 1346 nm. The optical fiber is coupled free-space and impinges under a 10° angle from the normal. To ensure the highest overlap of the impinging light and the *p–i–n* structure, the position of the optical fiber was optimized (distance to surface ~2 μm). Measurements are performed on cross-like structures since the contact design fitted well with the RF probes setup (see Supplementary Note 4). The voltage is applied between contacts 1 and 4 as indicated in Supplementary Fig. 2b. A typical DUT is depicted in Supplementary Fig. 2d. Using a vector network analyzer (VNA) the scattering parameter *S*_21_ of our devices is measured from 100 MHz to 30 GHz.

### Reporting summary

Further information on research design is available in the Nature Research Reporting Summary linked to this article.

## Supplementary information

Supplementary Information

Reporting Summary

## Data Availability

The authors declare that the data supporting the findings of this study are available with the paper and its Supplementary Information files. The data that support the findings of this study are available from the corresponding author upon reasonable request.
