# Supplementary material for: High-speed III-V nanowire photodetector monolithically integrated on Si
Source: Nat Commun. 2020 Sep 11;11:4565. doi: 10.1038/s41467-020-18374-z (PMC7486389; doi:10.1038/s41467-020-18374-z)
Supplement: Supplementary file 1 — Supplementary Information [file 41467_2020_18374_MOESM1_ESM.pdf]

Supplementary Information

High-speed III-V nanowire photodetector monolithically integrated on Si

Svenja Mauthe[1], Yannick Baumgartner[1], Marilyne Sousa[1], Qian Ding[2], Marta D. Rossell[1,3], Andreas Schenk[2], Lukas Czornomaz[1], and Kirsten E. Moselund[1]✉

[1] *IBM Research – Zurich, Säumerstr. 4, 8803 Rüschlikon, Switzerland*

[2] *Department of Information Technology and Electrical Engineering, Integrated Systems Laboratory, ETH Zurich, Gloriastr. 35, 8092 Zurich, Switzerland*

[3] *Electron Microscopy Center, Empa, Swiss Federal Laboratories for Materials Science and Technology, Überland Str. 129, 8600 Dübendorf, Switzerland*

✉ email: kmo@zurich.ibm.com

Supplementary Figures

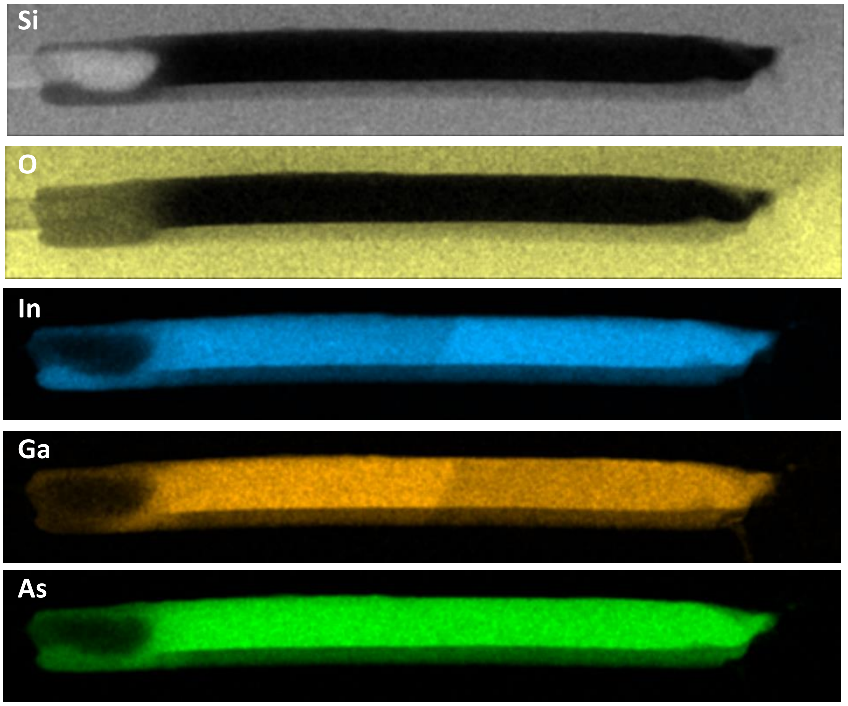

Supplementary Figure 1 **Detailed EDS analysis of grown nanostructure.** EDS maps of Si, O, In, Ga, and As. The EDS maps of Si and O reveal the Si seed on the left-hand side.

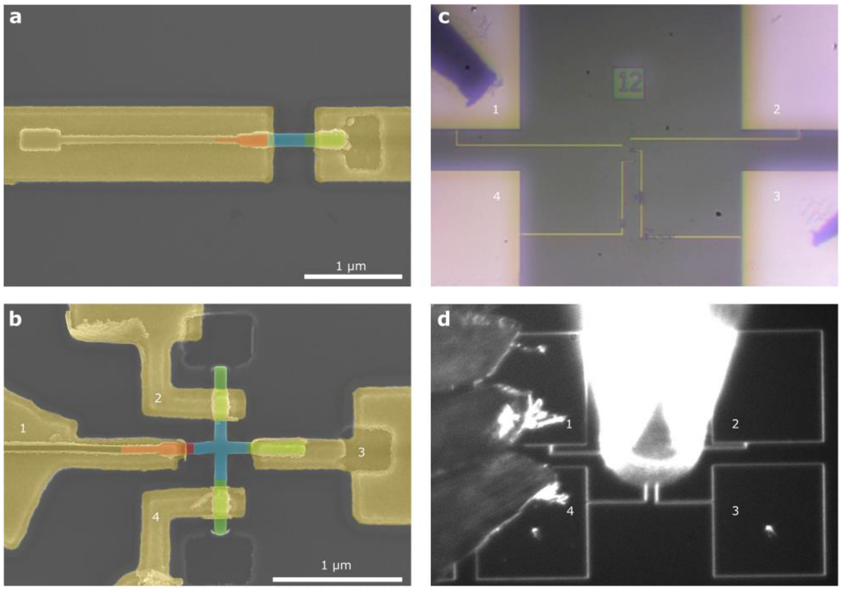

Supplementary Figure 2 **SEM images of fabricated devices.** False colored SEM images of a contacted *p-i-n* nanowire (a) and a *p-i-n* Hall cross bar structure (b). (c) DUT for PL, EL, and detection measurements. (d) DUT for high-speed measurements. RF-measurements are limited to current measurements between contact 1 and 4 due to the contact design.

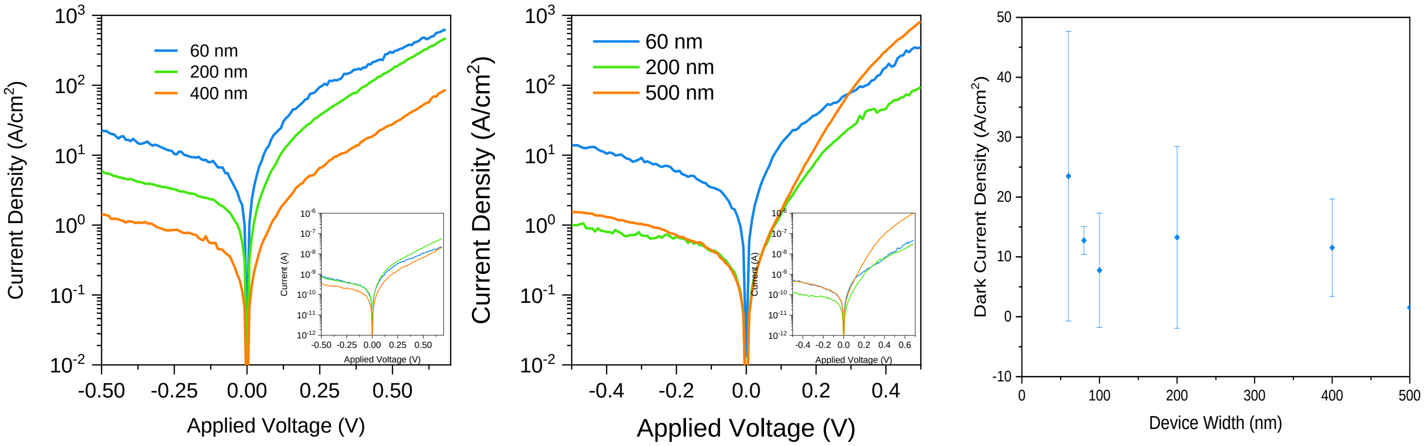

Supplementary Figure 3 **Electrical characterization.** (a) Current density versus applied voltage of devices with which the spectral response was measured. The inset depicts the current versus applied voltage of the devices plotted from 10^{-12} A to 10^{-6} A. (b) Current density versus applied voltage of devices with which the RF response was measured. The inset depicts the current versus applied voltage of the devices plotted from 10^{-12} A to 10^{-6} A. The results of these devices in (a) and (b) are presented in the paper. (c) Statistical overview of dark current densities measured for multiple devices of different widths.

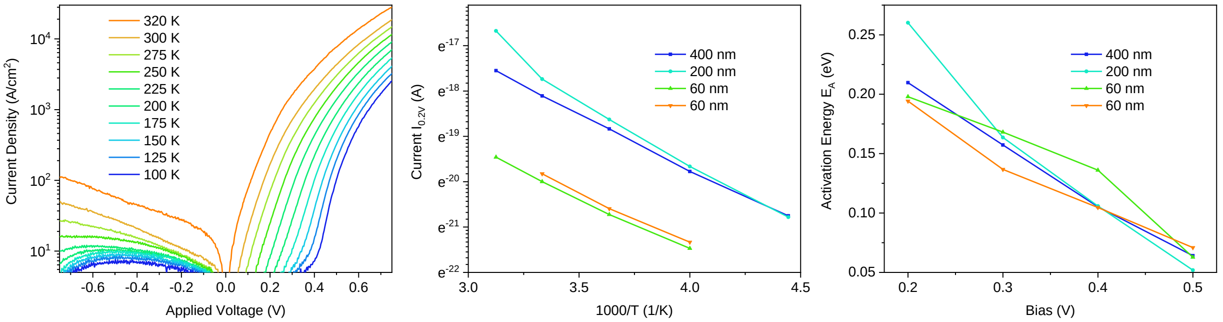

Supplementary Figure 4 **Temperature dependent electrical characterization.** (a) I-V curves of a 200 nm wide device at different temperatures from 100 K to 320 K. (b) Measured current at 0.2 V forward bias versus 1,000 T^{-1} for devices of different widths. The 200 nm curve contains data obtained from the measurement in (a). (c) Activation energy obtained using equation (2) and data from (b) for different devices widths and applied forward voltages.

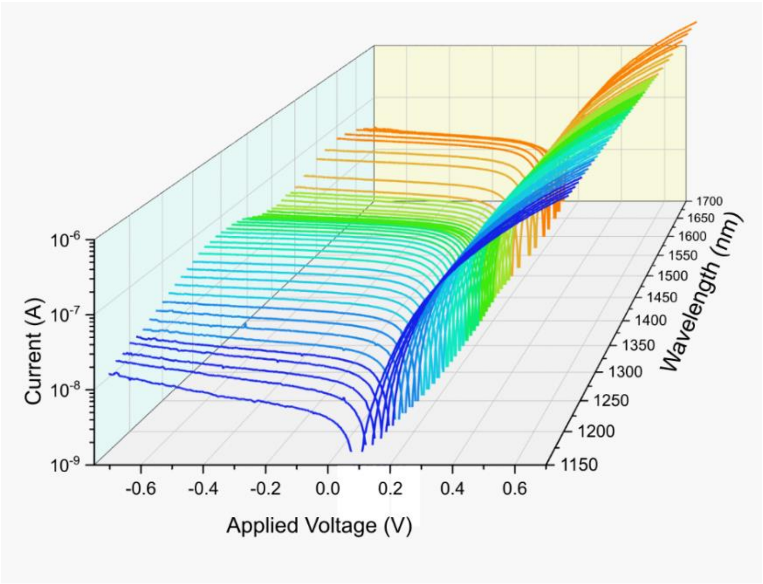

Supplementary Figure 5 **Spectral response of *p-i-n* photodetector.** Measured I-V curves for different incident wavelengths of the 200 nm wide device. The measurement is performed using a ps-pulsed supercontinuum laser.

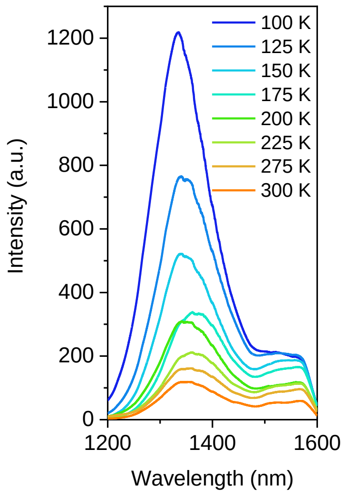

Supplementary Figure 6 **PL response at different temperatures.** The optical pump fluence is the same for all temperatures (~ 20 pJ pulse^{-1}).

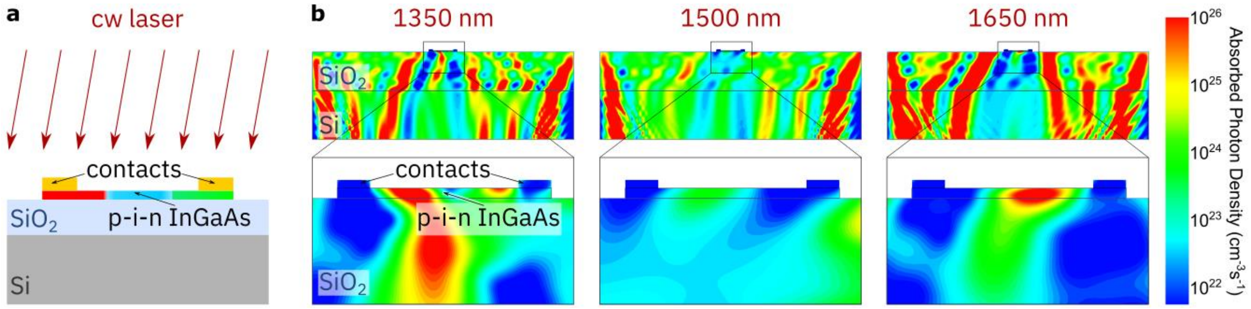

Supplementary Figure 7 **Simulated absorbed photon density.** (a) Sketch of the simulation setup. (b) Simulated absorbed photon flux density for different wavelengths. The upper images depict the simulation results of the entire simulated area. The lower images depict a close-up of the devices. Results in (b) are drawn to scale.

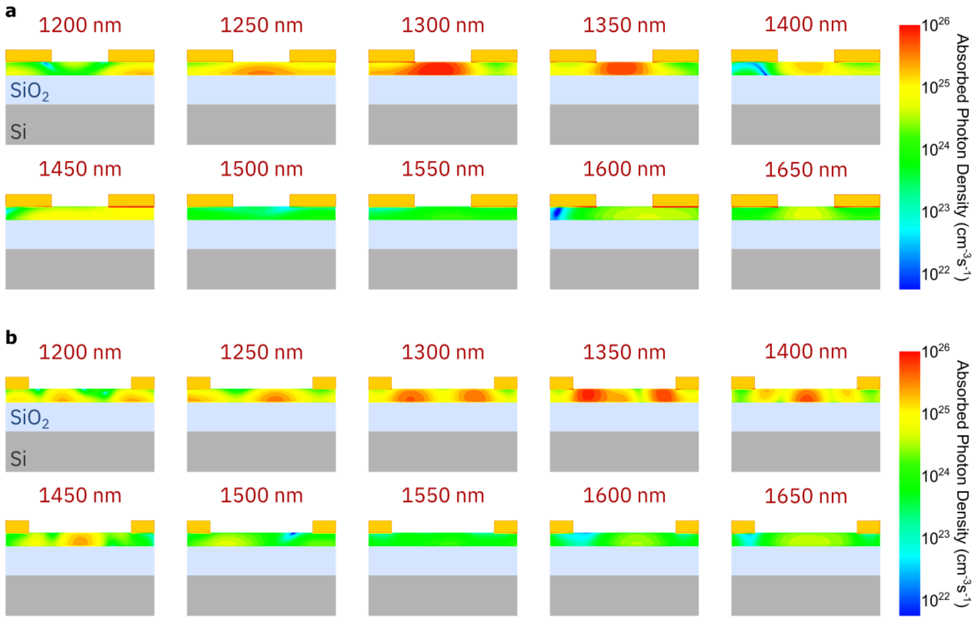

Supplementary Figure 8 **Simulated absorbed photon density for varying distance between metal contacts.** (a) Simulated absorbed photon density for a 60 nm thick device with distance between metal contacts of 400 nm. (b) Simulated absorbed photon density for a 60 nm thick device with distance between metal contacts of 900 nm. The scale in y-direction is enhanced by 2 for better visibility for both (a) and (b). The thickness of the BOX and Si layer are only indicated and do not match the values used in simulation.

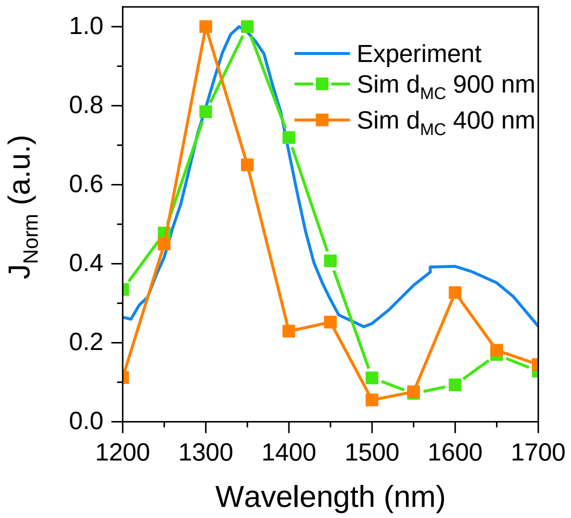

Supplementary Figure 9 **Comparison of measured and simulated normalized current density.** The measured device is 200 nm wide.

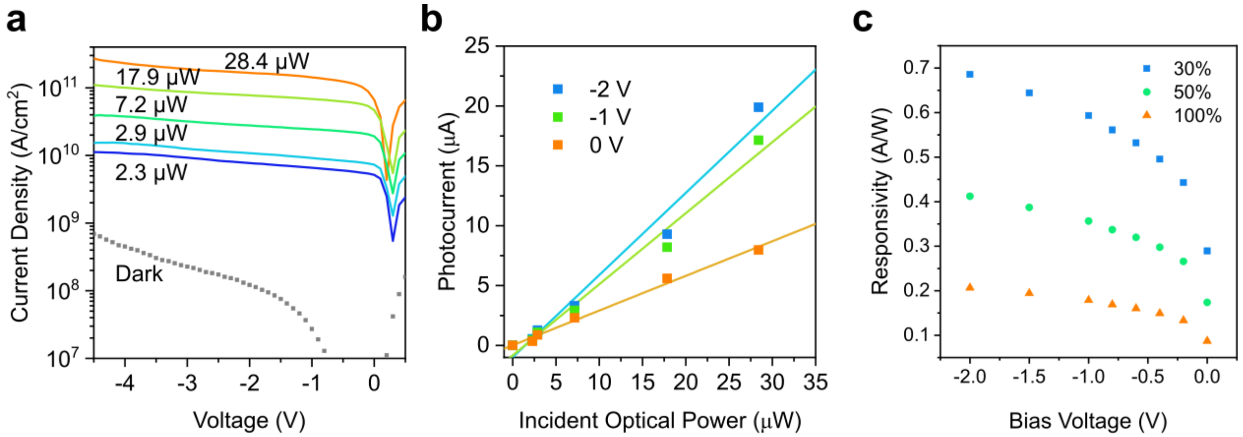

Supplementary Figure 10 **Characterization of responsivity.** (a) I-V curves for different incident optical powers (1346 nm cw laser) of a 200 nm wide device. (b) Photocurrent plotted versus the incident optical power for different applied reverse bias of the device in (a). The dots represent measured data, the lines a linear fit to determine the responsivity. (c) Responsivity versus applied voltage for different percentages of active devices areas compared to the total area. For a device with an active device length of 30 % of the total physical length of the III-V nanowire, it is assumed that only carriers generated in the 300 nm long intrinsic region of the 1 μm long device contribute to the current. This curve results from the fit in (b). If a larger fraction of the physical device area is taken into account, the responsivity decreases. The responsivity increases for a higher reverse bias.

Supplementary Tables

Supplementary Table 1 Molecular flow ratios for different growth sections during MOCVD growth of the *p*-*i*-*n* InGaAs structures.

	InAs seed	*p*-InGaAs	*i*-InGaAs	*n*-InGaAs
V/III	45	27	27	27
DeZn/(TMIn+TEGa)		0.41		
Si_2H_6/(TMIn+TEGa)				$2.07\ 10^{-3}$

Supplementary Note 1 – MOCVD Growth

The MOCVD growth is performed on 2 × 2 cm chips for convenience, however previous work demonstrated the successful TASE growth on 4” wafers [S. Lee et al., "High Performance InGaAs Gate-All-Around Nanosheet FET on Si Using Template Assisted Selective Epitaxy," 2018 IEEE International Electron Devices Meeting (IEDM)]. Before growth, the sample is cleaned thoroughly by performing a wet clean in heated piranha (70°C). This ensures a clean SiO_2 template and prevents parasitic nucleation. After the clean and shortly before loading the sample into the MOCVD reactor, a 20 s DHF dip is performed to remove the native oxide on the Si seed in the empty SiO_2 template. The sample is then instantly loaded into the growth chamber and put in vacuum. Any water residues are desorbed during a 5 min heating step at 750°C under TBA (50 sccm). This step is not necessary but ensures that any residues from the DI water are removed before the start of the III-V growth. Fig. 2 (b) in the manuscript depicts a schematic of the MOCVD growth procedure. After the initial anneal, the TBA flow is increased to 80 sccm for 2 min to ensure an As-terminated surface. Afterwards, a short InAs (precursors: TMIn, TBA (flow: 40 sccm)) (~ 20 nm) is grown which selectively nucleates at the Si interface. The InAs region facilitates the subsequent InGaAs nucleation. Next, InGaAs is grown using TMIn, TBA, and TBGa precursors with a V/III ratio of 27 and an TBA flow of 80 sccm. In-situ *p*- or *n*-doping is achieved by adding dopants. In this work, DEZn and Si_2H_6 are added as *p*- and *n*-dopants respectively. Supplementary Supplementary Table *1* depicts the different molecular flow ratios during MOCVD growth. At the end of the growth, the temperature is ramped down. Due to the growth in an oxide template, no post processing is required. However, if the crystal grew out of the template, it can be removed by performing a manual polishing process.

Supplementary Note 2 – Doping

Diethylzinc (DEZn) and disilane (Si_2H_6) precursors were introduced in-situ during the growth of the InGaAs homojunction to achieve *p*- and *n*-type doping, respectively. To quantify the doping concentration

in the *p*- and *n*-doped regions, separate individually *p*- and *n*-doped structures are fabricated exhibiting comparable dimensions as the final *p*-*i*-*n* structures. By measuring the Hall mobility, the doping concentration can be determined. Doping concentrations of ~ 3×10^{18} cm^{-3} and ~ 2×10^{19} cm^{-3} were found for *p*- and *n*-doped regions, respectively. These values, however, are measured in separate *p*- and *n*-structures and might therefore, vary slightly from the doping achieved in a *p*-*i*-*n* homojunction. The contacts on *n*-type InGaAs were found to be ohmic, on *p*-type non-ohmic, Schottky-type with a barrier height of ~ 200 meV as doping incorporation is lower in the *p*-doped region. The barrier height was experimentally measured as described in Supplementary Notes 6. The existence of a small barrier did not prevent high-speed operation.

Supplementary Note 3 – Material Characterization

Material characterization was performed using scanning electron microscopy (SEM), scanning transmission electron microscopy (STEM), and energy dispersive X-ray spectroscopy (EDS) on a focused ion beam prepared lamella containing a NW cross-section (see Fig. 3 in paper). Additional to the EDS elemental maps of As, In, and Ga depicted in the paper, Si and O elemental maps were obtained. Supplementary Figure 1 depicts all elemental maps, Si, O, In, Ga, and As. The Si seed is clearly visible on the left-hand side of the structure. After the Si seed, the width of the structure increases from the initial 60 nm width of the Si seed to 200 nm for this device. This leads to a short region in which all elements are overlapping the transmission EDS scan.

Supplementary Note 4 – Device Geometry

Supplementary Figure 2 (a) and (b) show two different device geometries fabricated in this work, a *p*-*i*-*n* nanowires and *p*-*i*-*n* Hall crosses, respectively. Device widths vary from 60 nm to 500 nm for PL, EL, detection, and high-speed measurements. EL measurements are performed on devices with 1,000 nm width.

Supplementary Figure 2 (c) depicts typical device under test (DUT) for PL, EL, and detection measurements. Details on the measurement setup can be found in the Method section. Supplementary Figure 2 (d) depicts a typical DUT in the high-speed measurement setup. Due to the contact design, high-speed measurements are performed on Hall cross bar structures between contact 1 and 4 as indicated in **Error! Reference source not found.**(b) and (d). They contain the same *p*-*i*-*n* profile, and repeated experiments show that the difference in geometry and therefore, electrical lay-out does not impact measured results.

Supplementary Note 5 – Dark Current

The dark current I_{dark} was measured in an I-V setup using a Keysight B1500A parameter analyzer. The voltage was swept from -0.5 V to +0.7 V. Device with different widths were measured. Supplementary Figure 3 (a) depicts I-V curves from the devices presented in the paper. Supplementary Figure 3 (b) shows a statistical distribution of the dark current density versus device width measured in a total of 29 devices. We observe a variation in dark current density in particular for smaller geometries. This may either be related to local variations in the contact fabrication or interface states and considering the small scale of the devices some variation is not surprising.

Supplementary Note 6 – Temperature-dependent Measurements – Activation Energy

To determine the barrier height, I-V curves at different temperatures are measured. Supplementary Figure 4 (a) depicts I-V curves of a 200 nm wide device at different temperatures. Due to a relatively high noise level in this setup, currents below ~5 A cm^{-2} are neglected. The barrier height can be extracted from an Arrhenius plot using the following approximated equation:

$$I \sim e^{-\frac{E_A}{k_B T}}, \tag{1}$$

where I is the measured current, E_A the activation energy, k_B the Boltzmann constant, and T the temperature. By plotting ln(I) over T as, the activation energy can be determined using:

$$\ln(I) \sim -\frac{E_A}{k_B}\frac{1}{T} \ . \tag{2}$$

Supplementary Figure 4 (b) shows the measured current at 0.2 V bias for different devices plotted via 1,000 T^{-1}. The resulting activation energy is plotted in Supplementary Figure 4 (c).

Supplementary Note 7 – Spectral Response

The spectral response of the devices is measured using a ps-pulsed tunable supercontinuum laser between 1200 nm and 1700 nm (78 MHz repetition rate). The light in focused onto the structures using a 10× objective (details in Method section). The bias voltage is swept between -0.75 V and 0.7 V and the current through the device is measured. Supplementary Figure 5 depicts the incident wavelength dependent measured IV curves for a 200 nm wide device (see paper Fig. 4). The values given in the paper for the spectral response are those recorded at a bias of -0.5 V.

Supplementary Note 8 – Photoluminescence spectroscopy at different temperatures

PL spectroscopy was performed at temperatures ranging from 100 K to 300 K. The sample was optically excited from the top using a ps-pulsed laser source around 720 nm (78 MHz repetition rate) and a 10× objective (details in Method section). Supplementary Figure 6 depicts the PL response of *p-i-n* structures measured at different temperatures. With decreasing temperatures, the PL signal increases and shifts towards the blue.

Supplementary Note 9 – Electrooptical Simulations

Coupled 2D opto-electrical simulations were employed to gain further insights into observed device behavior, especially the spectral response (details see Method section). First, 2D Finite-Difference-Time-Domain (FDTD) optical simulations using *Sentaurus EMW Solver* are performed to produce an absorbed photon density profile in the entire *p*-*i*-*n* structure. This absorbed photon density profile will then be taken as input in the following electrical transport simulation using *Sentaurus Device* to determine the device current at a given excitation wavelength.

Supplementary Figure 7 (a) depicts the simulated device and the simulation area. The simulated *p*-*i*-*n* devices are 60 nm high and 1.3 µm long. The InGaAs composition is assumed to be constant at 55 % indium to keep the simulation time reasonable. The doping concentration takes the same value as experiments (see Supplementary Notes 1). A monochromatic light source is placed at 2 µm above the device illuminating the device under an angle of 10° to mimic the free-space excitation (see Supplementary Figure 7 (a)). The experimental measurements of the spectral response have been performed with a pulsed supercontinuum laser. Therefore, the measured current in the *p*-*i*-*n* structures represents an average current over the incoming pulsed light (50 ps pulse width every 12.5 ns). The experimental value of the responsivity, on the other hand, is calculated based on experimental data using a CW laser at 1350 nm in a different set-up, which is also the one used for the high-speed measurements. We assume in all simulations a CW pump source of an average power corresponding to the experiment. To best reflect geometrical effects of experimental setup on optics, the metal contacts and SOI substrate are included.

Supplementary Figure 7 (b) shows the simulated absorbed photon density of the full simulation space including SOI wafer, III-V devices, and metal contacts with 900 nm spacing at 1350 nm, 1500 nm and 1650 nm. Multiple reflections of the incident light at the Si/SiO_2 interface are visible. These reflections are more pronounced for certain wavelengths and lead to an increase in absorbed photon density in the active III-V material. Supplementary Figure 8 (a) depicts the absorbed photon density in the *p*-*i*-*n* region. The simulation reveals an increase in absorbed photon density around 1350 nm and 1650 nm. Using the received

absorbed photon densities, the spectral response can be simulated. Supplementary Figure 9 depicts the simulated spectral response with two peaks at 1350 nm and 1650 nm. The simulated spectral response fits very well with the experimental curve, confirming the experimental measurements, and suggesting that the measured spectral response is impacted by the device geometry.

To further investigate the geometrical effects on the spectral response of the device, an additional simulation is performed where the distance between metal contacts is reduced to 400 nm (see Supplementary Figure 8 (b) and Supplementary Figure 9). The resulting simulated spectral response curve shows two peaks which are slightly shifted to lower wavelengths.

Supplementary Note 10 – Responsivity and external quantum efficiency

To determine the responsivity of the photodetectors, IV curves under different illumination powers were measured using a continuous wave laser at 1346 nm and a value for the incident radiation power is calculated. The measurements were performed in the RF setup. The optical fiber (NA 0.14, MFD 5.85 μm) impinges with a 10° angle to the normal direction of the wafer and is as close as 2 μm to the surface. Consequently, an illumination spot with a radius of $r_L \sim 3$ μm and a total power of P_0 impinges onto the sample. The calculation of the incident optical power is performed under the assumption of light absorption in the intrinsic region. For a photodetector with 200 nm width, we assume that the light is absorbed in the 300 nm intrinsic region, hence resulting in an active device area of 200 × 300 nm^2. Assuming a gaussian field profile for the laser light, the incident optical power P_{in} on the active device are can be calculated via

$$P_{\mathrm{in}} = P_0 \cdot \left(1 - \mathrm{e}^{-2\left(\frac{r_{\mathrm{D}}}{r_{\mathrm{L}}}\right)^2}\right) . \quad (3)$$

Here r_D is the relative device radius received from the device area. In this calculation we assume a maximum absorption, based on the spectral response this assumption most likely leads to an underestimation of the value of the responsivity as the increased response at shorter wavelengths points at the fact that a substantial

fraction of the incident light passes through the only 60nm thick structure. On the other hand, internal reflection as well as reflection from sidewalls and contacts would lead to multiple passages of the light which would boost the responsivity. The responsivity is calculated via

$$R = \frac{I_{\mathrm{ph}}}{P_{\mathrm{in}}} \tag{4}$$

with I_{ph} being the measured photocurrent at the incident power P_{in}. The quantum efficiency can be calculated via

$$\eta_{\mathrm{e}} = \frac{h\nu}{eP_{\mathrm{in}}} I_{\mathrm{ph}} \tag{5}$$

The measured devices show responsivities up to 0.68 A W^{-1} at -2 V reverse bias. The responsivity is highly bias dependent as it depends on the magnitude of the electric field across the junction. Supplementary Figure 10 (c) shows R as a function of bias.

The calculated values for the responsivity are highly depends on the active device area. In an ideal device, only carriers generated in the intrinsic region would contribute to the measured current. However, in a real device carries generated in the *p*- and *n*-regions can also contribute to the current. To quantify the impact of this factor on the responsivity, the active device area in equation (3) is varied. Supplementary Figure 10 (c) depicts the calculated responsivities assuming different percentage of the semiconductor area in the active region. The smaller the area included in this calculation the higher the obtained responsivity. In our case the physical *i*-region length corresponds to 30 % of the total device area hence this is the maximum obtainable value. If instead we would choose to include photons absorbed in the entire *p*-*i*-*n* device area, the responsivity results in a lower value of 0.2 A W^{-1}. The reality most likely lie somewhere in between those two extremes, but closer to the 30%.
